# Energetics of Floor Gymnastics: Aerobic and Anaerobic Share in Male and Female Sub-elite Gymnasts

**DOI:** 10.1186/s40798-021-00396-6

**Published:** 2022-01-10

**Authors:** Sebastian Kaufmann, Martin Ziegler, Jana Werner, Christine Noe, Richard Latzel, Stefan Witzany, Ralph Beneke, Olaf Hoos

**Affiliations:** 1 Centre for Sports and Physical Education, Faculty of Human Sciences, Julius-Maximilians-University Wuerzburg, Am Hubland/Sports Centre, 97074 Wuerzburg, Germany; 2 Faculty of Applied Healthcare Sciences, Deggendorf Institute of Technology, Deggendorf, Germany; 3 Department Medicine, Training and Health, Institute of Sports Science, Philipps-University Marburg, Marburg, Germany

**Keywords:** Energy contribution, PCr-LA-O_2_, Artistic gymnastics, Sex differences, Performance, Anaerobic

## Abstract

**Background:**

Artistic gymnastics is a popular Olympic discipline where female athletes compete in four and male athletes in six events with floor exercise having the longest competition duration in Women’s and Men’s artistic gymnastics (WAG, MAG). To date no valid information on the energetics of floor gymnastics is available although this may be important for specific conditioning programming. This study evaluated the metabolic profile of a simulated floor competition in sub-elite gymnasts.

**Methods:**

17 (9 male, 8 female) sub-elite gymnasts aged 22.5 ± 2.6y took part in a floor-training-competition where oxygen uptake was measured during and until 15 min post-exercise. Additionally, resting and peak blood lactate concentration after exercise were obtained. The PCr-LA-O_2_ method was used to calculate the metabolic energy and the relative aerobic (*W*_AER_), anaerobic alactic (*W*_PCr_) and anaerobic lactic (*W*_BLC_) energy contribution. Further, the athletes completed a 30 s Bosco-jumping test, a countermovement jump and a drop jump.

**Results:**

The competition scores were 9.2 (CI:8.9–9.3) in WAG and 10.6 (CI:10.4–10.9) in MAG. The metabolic profile of the floor routine was mainly aerobic (58.9%, CI: 56.0–61.8%) followed by the anaerobic alactic (24.2%, CI: 21.3–27.1%) and anaerobic lactic shares (16.9%, CI:14.9–18.8%). While sex had a significant (*p* = .010, *d* = 1.207) large effect on energy contribution, this was not the case for competition duration (*p* = .728, *d* = 0.061). Relative energy contribution of WAG and MAG differed in *W*_AER_ (64.0 ± 4.7% vs. 54.4 ± 6.8%, *p* = .004, *d* = 1.739) but not in *W*_PCr_ (21.3 ± 6.1% vs. 26.7 ± 8.0%, *p* = .144, *d* = 0.801) and *W*_BLC_ (14.7 ± 5.4% vs. 18.9 ± 4.2%, *p* = .085, *d* = 0.954). Further no correlation between any energy share and performance was found but between *W*_PCr_ and training experience (*r* = .680, *p* = .044) and *W*_BLC_ and competition level (*r* = .668, *p* = .049).

**Conclusion:**

The results show a predominant aerobic energy contribution and a considerable anaerobic contribution with no significant difference between anaerobic shares. Consequently, gymnastic specific aerobic training should not be neglected, while a different aerobic share in WAG and MAG strengthens sex-specific conditioning. All in all, the specific metabolic share must secure adequate energy provision, while relative proportions of the two anaerobic pathways seem to depend on training and competition history.

## Key Points

- The metabolic profile of floor gymnastics is strongly fueled aerobically, but anaerobic sources are highly relevant. This is the case in both Women’s and Men’s artistic gymnastics.
- Specific metabolic share during a floor competition may represent an “admission ticket” function to the sport and must secure an adequate energy provision.
- Sex has a significant influence on relative energy contribution in a simulated floor competition, which at least partly strengthens the traditional practice of sex-specific conditioning in artistic gymnastics.
- Sex-specific floor routines are recommended in training programs to enhance gymnastic-specific endurance.

## Background

Artistic gymnastics is a traditional, popular spectator sport and part of the Olympic Games since 1896 [1]. In artistic gymnastics women compete in four and men in six different events, respectively [2, 3]. In the past there has been a considerable development of difficulty in the routines on various apparatus [4]. In order to compete in artistic gymnastics both women and men need high levels of strength, flexibility and coordination and technical ability is seen as the main performance factor [5, 6].

Although exercise durations vary strongly from approximately 5 s in the vault to maximally 90 s on the floor in women’s artistic gymnastics (WAG) and up to 70 s on the floor in men’s artistic gymnastics (MAG) it has been assumed that energy supply and energy share may not play a decisive role for performance in artistic gymnastics [6]. In addition to the sex-specific competition duration judging between WAG and MAG varies according to FIG regulations. The higher focus on gymnastic transitions and fluency of the routine in WAG results in a more continuous movement pattern throughout the competition in WAG compared to MAG [2, 3]. However, this may also be the case because metabolic measurements during artistic gymnastics are difficult to conduct and there is only limited data available on the exercise intensity of WAG and MAG [7]. The limited data that is available points toward submaximal metabolic intensities for floor gymnastics. For male elite gymnasts maximal heart rate values of 186 ± 11 bpm and a delta in blood lactate concentration (∆BLC) of 5.19 mmol∙l^−1^ are reported as response to a simulated floor competition [5]. For female gymnasts high heart rate values [8] and blood lactate concentrations (BLC) of 7–8.5 mmol∙l^−1^ have been reported after floor routines [9, 10]. Furthermore, oxygen uptake values (VO_2_) up to 40 ml∙kg^−1^∙min^−1^ have been estimated for women´s floor routines [7]. There have also been attempts to estimate the relative taxation of energy systems in artistic gymnastics. For floor competitions rather rough estimations yielded that the ATP-PCr System is taxed by 100% in both WAG and MAG, that anaerobic glycolysis is taxed up to 80–90% in WAG and 60–70% in MAG and that the aerobic system by 20–30% in both WAG and MAG [5, 7, 10]. The foundation for these estimates based on blood lactate, heart rate and oxygen consumption values in relation to maximal attainable values is unclear and may not consider the complex interactions of the energy systems during intense exercise [11, 12]. Further, these estimates may lead to a misinterpretation of the importance of the respective energy system for the performance in floor competitions. Additionally, it is unclear how competition level and element difficulty influence metabolic demand and relative energy contribution in artistic gymnastics.

Research in ballet and figure skating that also focus on technical ability and involve short intense movements has shown that aerobic energy supply was predominant, which is in contrast to earlier, traditional assumptions [13, 14]. Moreover, the results of the latter studies highlight the importance of anaerobic alactic energy contribution and point toward a possible relation between technical ability and relative energy contribution. According to different energy estimation models for maximal intensity exercise over 70 to 90 s relative aerobic energy contribution would likely be in a range between 40 and 60% [11]. Considering the above quoted heart rate and BLC data the intensity of a floor routine may only be near maximal. Yet, floor gymnastics certainly involves more muscle mass than running or cycling exercise on which the estimation models in Gastin [11] are based. Moreover, due to the high importance of anaerobic energy contribution in artistic gymnastics it would be interesting to know the relative anaerobic lactic and anaerobic alactic fractions to enhance the foundation of sport-specific exercise prescription. This would be important to ensure that athletes compete at submaximal intensities to better avoid technical mistakes and/or prevent injuries. In addition, the competition duration and the rating of the routines by the judges are different in WAG and MAG [2, 3], consequently possible differences in energy share should be evaluated.

Therefore, the aim of this study was twofold: firstly, to provide the first full energetic profiles of a floor-training-competition in WAG and MAG and secondly to compare the energy share of WAG and MAG. We hypothesized that (I) an aerobic energy contribution of at least 50% and a substantial anaerobic alactic energy share would occur in a simulated floor gymnastic competition in both sexes and (II) that the energy share would differ between WAG and MAG with a less pronounced reliance on aerobic energy in MAG.

## Methods

### Participants

Eight female (age: 21.6 ± 2.8yrs, height: 161 ± 4 cm, weight: 60.3 ± 6.8 kg, ~ 8 h training per week) and nine male (age: 23.2 ± 2.5yrs, height: 175.2 ± 7 cm, weight: 71.3 ± 6.7 kg, ~ 8 h training per week) sub-elite gymnasts of the University`s artistic gymnastics team participated in the study. All athletes were free from any medical issues and were informed about reasons and risks of the measurements. All subjects signed informed consent, the study was approved by the institutional ethics committee of the Philipps-University Marburg (AZ-3-12-18) and carried out in accordance with the standards of ethics outlined in the Declaration of Helsinki.

### Procedure

All participants took part in a floor competition (FC) with metabolic measurements in a gym. The floor competition which was conducted on an official artistic gymnastics floor (Spieth Gymnastics GmbH, Altbach, Germany) was carried out like an official competition to mimic realistic conditions as much as possible. Due to the measurements, i.e., breath-by-breath spirometry it was not possible to conduct the experiment during an official competition. The routine for the FC was developed by the gymnastics team coach with assistance of an experienced athlete and the laboratory manager in order to ensure an officially valid but also secure routine for athletes and equipment. Finally, the developed routine was overseen and approved by an experienced, official judge of the Bavarian Gymnastics Federation. The only modification compared to a regular floor competition concerned the difficulty of the artistic elements: As each athlete normally performs an individual exercise with elements appropriate to his or her ability, the exercise difficulty was reduced in a standardized manner. Both exercise difficulty and the standardized reduction in difficulty were applied as laid down in international competition rules of the “Code de Pointage” (CdP)(FIG, Lausanne, Switzerland) [2, 3]. Prior to the test, all athletes were given the chance to train for the FC routine for an appropriate amount of time during the team training and to accommodate to wearing the spirometry equipment during the routine. On the day of the simulated FC all athletes competed in a randomized order and under similar conditions as in a regular competition. All subjects prepared by themselves and used their individual pre-competition warm-up routine. Then, the subjects were precisely instructed to the test protocol, again. In order to calculate the metabolic profile for each athlete using the PCr-LA-O_2_ method [15] oxygen consumption (VO_2_) was continuously measured during the FC and until 15 min post-exercise using a portable breath-by-breath metabolic cart (Metamax 3B, Cortex Biophysik GmbH, Leipzig, Germany). The metabolic cart was calibrated before being attached to each athlete. From pre-tests we knew that data quality may be impaired by very hard impact forces during landing and by restricted breathing patterns. Therefore, two researchers independently checked the raw VO_2_ data for unphysiological breath-by-breath variations. In three cases high shock levels resulted in unphysiological bumps in the VO_2_ curve. In these cases (1 male, 2 female) the measurements were carried out again two days later. Moreover, before, immediately after the test and 1, 2, 3, 4, 5 and 7 min post-test [16], 20µL capillary blood were collected from the hyperemic ear lobe for enzymatic-amperometric blood lactate concentration determination (Biosen C-line, EKF-Diagnostik, Eppendorf, Germany). Additionally, HR was obtained continuously via a portable HR monitor (H10; Polar Electro Oy, Kempele, Finland). All athletes were filmed during their floor routine and the execution was independently evaluated by two official judges who strictly followed the international rules of the “Code de Pointage” (CdP) [2, 3]. The final score of FC was calculated as: final score = difficulty score + execution score, with difficulty score being predefined according to the CdP and execution score equaling 10,00—average withdrawal from the two judges.

In addition to the simulated competition, jumping ability and anaerobic power were assessed by a standardized drop-jump test from 45 cm height (DJ), a counter-movement jump (CMJ) [17] and a 30 s Bosco-Continuous-Jumping-Test (CJ30) [18]. For the DJ subjects were instructed to jump as high as possible while keeping the ground contact time as short as possible and the CJ30 was carried out exactly as described in the original study [18]. The jump tests were conducted three days apart from the simulated floor competition at a comparable time of the day to ensure that all athletes would be free from fatigue or delayed onset of muscle soreness. Again, all athletes were familiarized with the test procedures and the test order was assigned randomly. Before the jump tests a standardized warm-up protocol was performed to prepare the athletes for high intensity activity. During the warm-up intense movements did not last longer than 3 s, in order to avoid blood lactate concentration accumulation [19]. Ground contact time and flight time were measured for DJ, CMJ and CJ30 with photoelectric cell technology (Optojump Next, Microgate, Bolzano, Italy). Jump height (in cm) and jump power (in W∙kg^−1^) were calculated based on these values by the Optojump software, which can be considered a valid procedure [20]. Peak and mean power (in W∙kg^−1^) for CJ30 were calculated as described in Bosco, Luhtanen [18]. Additionally, before, immediately after CJ30, as well as 1, 2, 3, 4, 5 and 7 min post-test [16], 20µL capillary blood were collected from the hyperemic ear to assure determination of peak blood lactate concentration (BLC_peak_ in mmol∙l^−1^; Biosen C-line, EKF-Diagnostik, Eppendorf, Germany).

### Calculation of the Metabolic Profiles

The individual metabolic profiles for WAG and MAG were calculated using the PCr-LA-O_2_ method [15]. Consequently, metabolic energy (*W*_tot_) was calculated as the sum of the absolute aerobic (*W*_AER_), anaerobic lactic (*W*_BLC_) and anaerobic alactic (*W*_PCr_) shares:

$$W_{{{\text{tot}}}} = W_{{{\text{AER}}}} + W_{{{\text{BLC}}}} + W_{{{\text{PCr}}}}$$

and metabolic power (*P*_tot_) as *W*_tot_ divided by exercise duration:

$$P_{{{\text{tot}}}} = W_{{{\text{tot}}}} \div t$$

All energy shares were calculated in J∙kg^−1^ and are presented in absolute (J∙kg^−1^) and relative (% of *W*_tot_) numbers. *W*_AER_ in J∙kg^−1^ was calculated from VO_2_ above rest during FC, caloric equivalent, and body mass by using:

$$W_{{{\text{AER}}}} \left( {{\text{J}}\;{\text{kg}}^{ - 1} } \right) = {\text{VO}}_{2} \left( {{\text{ml}}\,{\text{kg}}^{ - 1} } \right) \cdot {\text{caloric}}\,{\text{equivalent}} \left( {{\text{J}}\,{\text{ml}}^{ - 1} } \right)$$

Since measurement of the resting VO_2_ before the tests may be difficult due to sympathetic arousal, the equivalent of VO_2_ in a standing position (4.5 ml∙kg^−1^∙min^−1^) was defined as the resting VO_2_ [21]. Due to increased muscle mass and lower body fat percentage when compared to female non-gymnasts of the same age [22, 23] this value was also applied for the modeling of the metabolic profiles in WAG. Accordingly, VO_2_ above rest during FC was calculated as the area under the curve of actual VO_2_ minus 4.5 ml∙kg^−1^∙min^−1^. Anaerobic lactic energy (*W*_BLC_) was determined from the highest change in blood lactate concentration (Net-BLC) and body mass by using:

$$W_{{{\text{BLC}}}} \left( {{\text{J}}\;{\text{kg}}^{ - 1} } \right) = \Delta BLC \left( {{\text{mmol}}\;{\text{l}}^{ - 1} } \right) \cdot O_{2} - {\text{lactate}}\,{\text{equivalent}} \left( {{\text{ml}}\;{\text{kg}}^{ - 1} \;{\text{mmol}}^{ - 1} \;l} \right) \cdot {\text{caloric}}\,{\text{equivalent}} \left( {{\text{J}}\,{\text{ml}}^{ - 1} } \right)$$

Assuming a distribution space of lactate close to 45% of the body mass, the O_2_-lactate equivalent is 3.0 ml∙kg^−1^∙mmol^−1^∙l [24]. A value of 20.9 J∙ml^−1^ was employed as caloric equivalent [11]. *W*_PCr_ was estimated based on the fast component of post-exercise oxygen uptake (VO_2PCr_) calculated from the latter and body mass by:

$$W_{{{\text{PCr}}}} \left( {{\text{J}}\,{\text{kg}}^{ - 1} } \right) = {\text{VO}}_{{{\text{2PCr}}}} \left( {{\text{ml}}\,{\text{kg}}^{ - 1} } \right) \cdot {\text{caloric}}\,{\text{equivalent}} \left( {{\text{J}}\,{\text{ml}}^{ - 1} } \right)$$

Due to the high exercise intensity a bi-exponential model:

$${\text{VO}}_{{{\text{2EPOC}}}} \left( {{\text{ml}}\,{\text{kg}}^{ - 1} } \right) = a \cdot e^{{\left( { - t \div \tau a} \right)}} + b \cdot e^{{\left( { - t \div \tau b} \right)}} + c$$

was used to fit the fast component of the post exercise oxygen uptake [15]. Then VO_2PCr_ (ml∙kg^−1^) was derived from the integral of the fast component using:

$${\text{VO}}_{{{\text{2PCr}}}} = a \cdot e^{{\left( { - t \div \tau a} \right)}}$$

To secure a high precision of our model the goodness-of-fit for the curve fitting process had to be r^2^ > 0.95.

### Statistical Analysis

Data-processing procedures and statistics were computed using SPSS 26 (IBM, Chicago, IL) and Origin 2019b (OriginLab, Northampton, MA). Kolmogorov–Smirnov testing and the Levene statistics for homoscedasticity were used to verify the normality of distribution. Differences in energy system contribution between WAG and MAG were tested using a two-way ANOVA (sex × energy system) with repeated measures on the second factor and Bonferroni post-hoc testing. Since FC time was significantly different an additional analysis of covariance was carried out with exercise time as a covariant. Additionally, a one-way ANOVA with repeated measurements was carried out to determine differences between energy systems within WAG and MAG. Differences between performance variables of WAG and MAG were tested by t-tests for independent samples. Statistical correlations between variables are indicated by Pearson’s *r*. All statistical tests were deemed to be significant at *p* ≤ 0.05 and effect sizes are shown as Cohen`s *d* and results are presented as means plus minus standard deviation and 90% confidence intervals (CI).

## Results

**Fig. 1:**
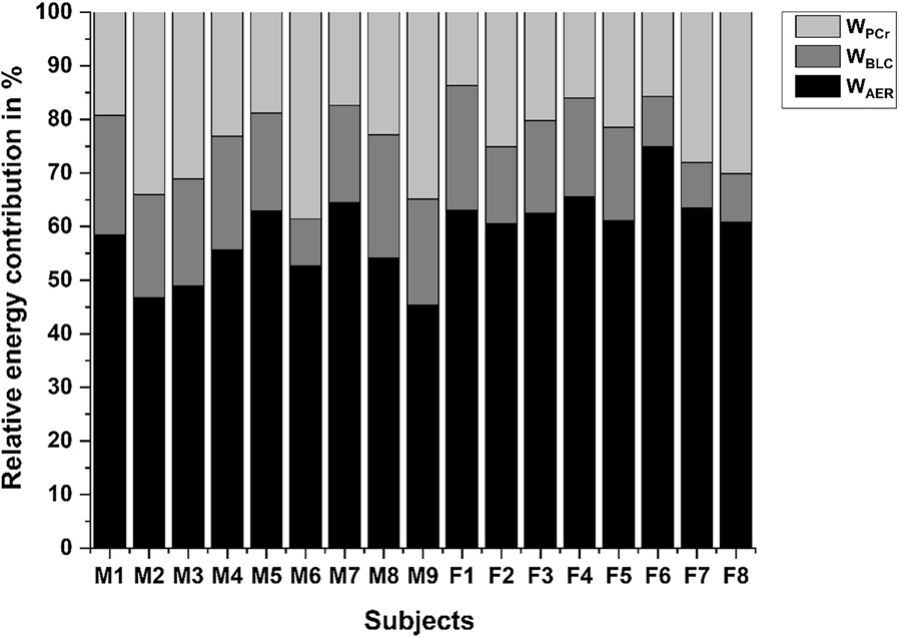
Individual, relative energy contribution of female and male subjects to the simulated floor competitions lasting 86 s in WAG and 72 s in MAG. Energy shares are shown as aerobic (*W*_AER_), anaerobic lactic (*W*_BLC_) and anaerobic alactic (*W*_PCr_)

The overall metabolic profile for the floor routines with data of men and women combined was predominantly aerobic: *W*_AER_ 58.9 ± 7.3% CI: 56.0–61.8%, *W*_PCr_ 24.2 ± 7.2% CI: 21.3–27.1%, *W*_BLC_ 16.9 ± 4.9% CI:14.9–18.8%. The respective individual profiles of relative energy contribution are displayed in Fig. 1. The metabolic profiles (WAG: *W*_AER_ 64.0 ± 4.7% CI:61.3–66.7%, *W*_PCr_ 21.3 ± 6.1% CI:17.8–24.8%, *W*_BLC_ 14.7 ± 5.4% CI:11.6–17.8%; MAG: *W*_AER_ 54.4 ± 6.8% CI:50.7–58.1%, *W*_PCr_ 26.7 ± 8.0% CI:22.3–31.1%, *W*_BLC_ 18.9 ± 4.2% CI:16.7–21.2) of the simulated floor competition (FC) as well as performance parameters from DJ, CMJ and CJ30 are shown in Table 1. There was a significant overall effect of sex (*F* = 5.447, *p* = 0.010, *d* = 1.207) on energy system and relative energy shares were significantly different from each other (F = 160.265, *p* < 0.001, *d* = 6.549). The relative energy contribution of WAG and MAG differed in *W*_AER_ (*F* = 11.3, *p* = 0.004, *d* = 1.739) but not in *W*_PCr_ (*F* = 2.4, *p* = 0.144, *d* = 0.801) and *W*_BLC_ (*F* = 3.4, *p* = 0.085, *d* = 0.954). Due to competition rules, the floor competition time was different between male and female gymnasts (71.6 ± 4.7 s CI: 68.0–73.6 s vs. 85.5 ± 2.0 s CI:84.6–87.8 s, *p* < 0.001, *d* = − 3.7), but FC time did not significantly affect relative energy contribution (*F* = 0.126, *p* = 0.728, *d* = 0.061). Moreover, when the data of women and men were pooled, significant correlations were found for bodyweight and peak power (PP) and mean power (MP) in CJ30 (*r* = 0.922, *p* < 0.001, *r* = 0.930, *p* < 0.001), DJ height (*r* = 0.610, *p* = 0.012) and CMJ height (*r* = 0.653, *p* = 0.006). Further, *W*_PCr_ (J∙kg^−1^) was significantly, positively correlated with relative MP in CJ30 (*r* = 0.647, *p* < 0.001) and with training experience in years (*r* = 0.680, *p* = 0.044), while *W*_BLC_ (J∙kg^−1^) was correlated with competition level (*r* = 0.668, *p* = 0.049).

**Table 1:** Metabolic profiles in simulated floor competition (FC) and performance parameters from DJ, CMJ and CJ30 (means and standard deviations)

	MAG	WAG	
**Floor competition**			
Floor competition time	71.6 ± 4.7 s	85.5 ± 2.0 s	*p* < *.001, d* = − *3.7*
Metabolic energy (*W*_tot_)	1562.55 ± 224.1 J∙kg^−1^	1556.58 ± 184.8 J∙kg^−1^	*p* = *.953, d* = *0.03*
Metabolic power (*P*_tot_)	21.9 ± 3.22 W∙kg^−1^	18.2 ± 2.7 W∙kg^−1^	p = .02, *d* = 1.3
Aerobic energy (*W*_AER_)	54.4 ± 6.8%	64.0 ± 4.7%	*p* = *.004, d* = *1.7*
Anaerobic alactic energy (*W*_PCr_)	26.7 ± 8.0%	21.3 ± 6.1%	*p* = *.144, d* = *0.8*
Anaerobic lactic energy (*W*_BLC_)	18.9 ± 4.2%	14.7 ± 5.4%	*p* = *.085, d* = *1.0*
**Drop jump**			
DJ ground contact time	0.17 ± 0.01 s	0.17 ± 0.02 s	*p* = *.948, d* = − *0.03*
DJ height	30.7 ± 5.3 cm	26.1 ± 3.5 cm	*p* = *0.068, d* = *0.9*
DJ power	48.9 ± 9.4 W∙kg^−1^	42.2 ± 4.9 W∙kg^−1^	*p* = *.236, d* = *0.6*
**Counter-movement jump**			
CMJ jump height	37.6 ± 3.9 cm	30.9 ± 1.3 cm	*p* = *.001, d* = *2.1*
**30 s continuous jumping test**			
Peak power (MP)	23.9 ± 2.6 W∙kg^−1^	20.5 ± 2.4 W∙kg^−1^	*p* = *.015, d* = *1.4*
Mean power (PP)	21.9 ± 3.6 W∙kg^−1^	16.9 ± 1.4 W∙kg^−1^	*p* = *.004, d* = *1.7*

**Fig. 2:**
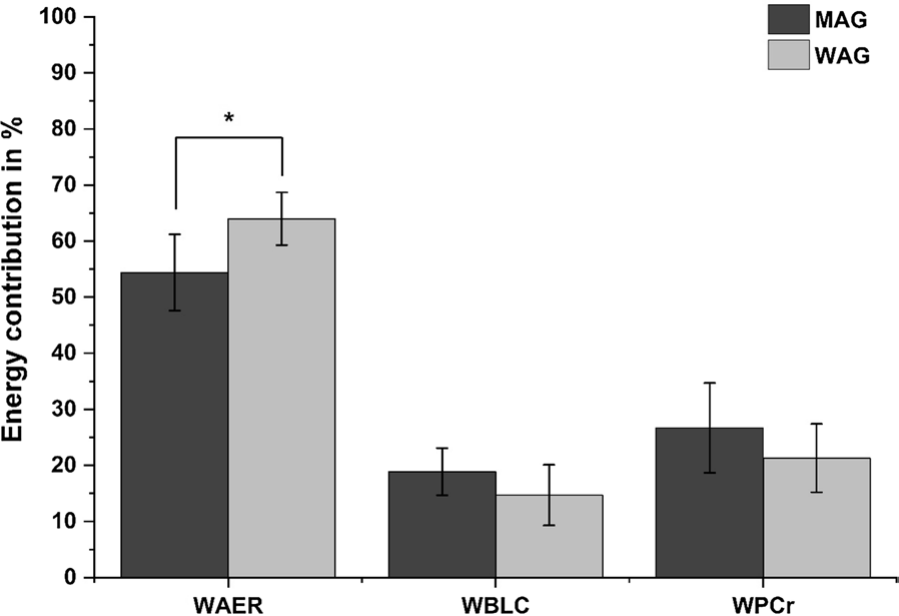
Relative energy contribution of WAG and MAG (means and standard deviations in percent). Energy shares are shown as aerobic (*W*_AER_), anaerobic lactic (*W*_BLC_) and anaerobic alactic (*W*_PCr_). * indicates a significant difference between *W*_AER_ in WAG and MAG

The female athletes reached a mean value of 9.2 ± 0.3 (CI:8.9–9.3) in FC. The athletes produced a *W*_tot_ of 1556.58 ± 224.10 J∙kg^−1^ (CI: 1426.25–1686.91 J∙kg^−1^) and *P*_tot_ of 21.9 ± 3.2 W∙kg^−1^ (CI: 16.64–19.79 W∙kg^−1^). Relative energy contribution differed significantly overall (*F* = 31.5, *p* = 0.001, *d* = 3.0) and between *W*_AER_ and *W*_PCr_ (*p* < 0.001, *d* = 7.904) and *W*_AER_ and *W*_BLC_ (*p* < 0.001, *d* = 5.198) but not between *W*_PCr_ and *W*_BLC_ (*p* = 0.115, *d* = 1.162)(Fig. 2). *W*_PCr_ in (J∙kg^−1^) was significantly, positively correlated with PP in CJ30 (*r* = 0.815, *p* = 0.026) and relative PP in CJ30 (*r* = 0.775, *p* = 0.041). Additionally, *W*_BLC_ (J∙kg^−1^) was significantly, positively correlated with time competing in the respective performance category / league (*r* = 0.765, *p* = 0.045). Finally, PP and MP in CJ30 was significantly and positively associated with bodyweight (*r* = 0.771, *p* = 0.042; *r* = 0.843, *p* = 0.017).

The male athletes in this study reached 10.6 ± 0.5 (CI:10.4–10.9) points in FC, individual results are presented. The athletes produced a *W*_tot_ of 1562.55 ± 224.1 J∙kg^−1^ (CI: 1426.26–1686.91 J∙kg^−1^) and *P*_tot_ of 21.9 ± 3.2 W∙kg^−1^ (CI: 16.64–19.79 W∙kg^−1^). Energy contribution differed significantly between the three systems (*p* = 0.037, *F* = 6.2). Moreover, *W*_AER_ differed significantly from *W*_PCr_ (*p* < 0.001, *d* = 3.751) and from *W*_BLC_ (*p* < 0.001, *d* = 6.298), while *W*_PCr_ and *W*_BLC_ (*p* = 0.064, *d* = 1.251) did not differ significantly. None of the energy contribution parameters was correlated with any performance parameters but both *W*_PCr_ (J∙kg^−1^) and *P*_tot_ (W∙kg^−1^) were significantly, positively correlated with training duration in years (*r* = 0.680, *p* = 0.044; *r* = 0.706, *p* = 0.034;). Additionally, the FC final score was significantly, positively correlated with time competing in the respective performance category / league (*r* = 0.702, *p* = 0.035).

## Discussion

This study provides the first energetic profile of floor gymnastics. With 58.9% of the total metabolic energy the floor routine was strongly fueled aerobically in this study. Besides the aerobic predominance, the anaerobic energy contribution is also highly relevant for the floor discipline with an anaerobic alactic share of 24.2% and an anaerobic lactic share of 16.9%. The slightly different competition time for male and female athletes did not influence the energetics significantly. Yet the energetic profile of WAG tends to be slightly more aerobic than in MAG which however still shows a mean aerobic contribution of over 50%. Thus, the data support our first hypothesis that a predominant aerobic energy share of at least 50% is present in floor gymnastics. Additionally, there was a significant and substantial effect of sex on relative energy system contribution confirming our second hypothesis. Therefore, while anaerobic training traditionally plays an important role for floor gymnastics sport-specific aerobic training should not be neglected [25]. Further, the role of sex-specific conditioning for WAG and MAG is at least partly supported by the results of this study.

### Energetic Profile of a Simulated Floor Competition: Discipline Specifics and Influencing Factors

Within an average FC duration of 85.5 ± 2.0 s the female athletes reached an FC score of 9.2 ± 0.3 which is representative for their sub-elite level. Frequently, athletes use almost the full 90 s available for floor routines. In this simulated competition it may have been the case that carrying the spirometry equipment led to a shorter execution time of static elements. All in all, the results show a relatively homogeneous level within the group. The male athletes reached a score of 10.6 ± 0.5 on average. Competition time slightly exceeded the official time limit for a floor routine with 71.6 ± 4.7 s. This may have been caused by the transition to rest and the simulative character of the competition.

The metabolic power *P*_tot_ was 18.2 W∙kg^−1^ in WAG and 21.9 W∙kg^−1^ in MAG. While the women’s *P*_tot_ was slightly lower than the one reported for a 120 s kayaking time trial [26], the men’s *P*_tot_ was lower than the 25.4 W∙kg^−1^ previously reported for 60 s simulated Judo matches but higher than the 18.9 W∙kg^−1^ for two minute simulated judo matches with elite and sub-elite judokas [27]. These data indicate high but still submaximal metabolic power values. Previous research on maximal intensity exercise showed values of 58 W∙kg^−1^ to 44 W∙kg^−1^ for 30 s all out tests [19] and ⁓27 W∙kg^−1^ for 200 s kayak sprints [28]. Relative metabolic energy in WAG was between the values (1163.6 J∙kg^−1^, 2455.6 J∙kg^−1^) shown for 40 s and 120 s kayaking time trials in women [26]. In MAG the relative metabolic energy was comparable to the energy for 60 s simulated Judo matches (1526.6 J∙kg^−1^) [27]. Therefore, both metabolic energy and metabolic power hint toward an overall submaximal taxation of the energy systems over time.

The largest part of that metabolic energy was supplied aerobically in this study. Regarding the exercise duration this has been expected for the pooled profile as well as for WAG and MAG and is confirmed by our data. All three values for the aerobic share are within the range for aerobic energy contribution that has been reported for maximal continuous exercise and highlights the importance of exercise duration for relative energy contribution also for sub- or near maximal intensity [11]. Especially in WAG the magnitude of the aerobic share was expectable due to exercise duration. Hence, the aerobic energy share in WAG was well in line with the range of the aerobic contribution in other sports with similar duration from grand adage ballet exercise in recreational/elite dancers (65%/77%, 210 s exercise duration) [13], over 120 s all-out kayaking (57.5%) to 226 s figure skating (74.1%) [14]. In MAG despite the shorter duration still 54% of the metabolic energy was supplied by the aerobic system. This is not as high as in WAG but rather equal to the total anaerobic share. However, this value is similar to those from 60 s simulated Judo matches, where the relative aerobic supply was 50% [27]. Consequently, this seems to be a realistic value for high to near-maximal intensity of 70 s duration involving a combination of intense short reactive and static elements interspersed with short phases of lower intensity where athletes are getting in position for the next high-intensity element. In this study the routines consisted of six paths with combinations approximately every seven seconds in MAG and four paths and intense elements such as a backwards salto or split leap every ten seconds in WAG. In between elements rated as very intense such as a salto or salto-combination connective elements or elements generating pre-acceleration such as roundoffs were performed, but also short static elements such as a standing scale. This activity profile points toward a fluctuating energy demand during FC. Therefore, we may assume that the aerobic energy system serves as a provider of a quasi-basic-energy rate over time, as the energy turnover of the aerobic system even at maximal rates would be far too small to provide energy for reactive jumps or static elements [29]. This is supported by submaximal VO_2_ values that have been reported for floor routines [25].

The high-intensity movements in floor gymnastics are likely to be fueled by anaerobic energy supply and consequently it has been assumed that gymnastics is an “anaerobic” sport [6, 7]. This is partly supported by our data that shows a general anaerobic contribution of 41% to floor exercise and even 46% for MAG and still 36% for WAG. Considering other research on sports with sub- or near maximal intensity and high focus on technical ability it becomes clear that the anaerobic share shows a relatively rapid decline with longer exercise durations [13, 14]. This is also in line with energy contribution in maximal intensity continuous exercise [11]. Therefore, although anaerobic contribution in this study was not predominant in floor gymnastics it can be regarded as a highly relevant energy source. Between the anaerobic alactic and anaerobic lactic share no significant difference was found neither in WAG nor MAG. Similarly, in figure skating and 120 s kayaking no clear difference between relative anaerobic alactic and anaerobic lactic energy contribution was found [14, 26]. However, in MAG the large effect size may indicate a possible substantial higher anaerobic alactic share. The latter is comparable to the anaerobic alactic share in 120 s judo matches (25.5%) but much lower than in 60 s judo matches (39.9%) [27]. However, in both WAG and MAG the large standard deviations may indicate large interindividual differences among the subjects. Also, among both anaerobic shares the standard deviations were comparably large with approximately one fourth and one third of the average value of anaerobic alactic and anaerobic lactic energy contribution. We may assume that both individual physiological and performance differences of the athletes, as well as training history may play a role in this regard. The magnitude of the anaerobic alactic share corresponds to approximately 12.6 mmol∙kg wet mm^−1^ in WAG and 14 mmol∙kg wet mm^−1^ in MAG and clearly indicate a submaximal taxation of the ATP-PCr system [30]. Likewise, the relative anaerobic lactic shares in WAG and MAG reflect a submaximal taxation of the anaerobic lactic energy system, which is supported by the BLC_peak_ values after the floor routine. While the BLC_peak_ values for WAG are roughly 2 mmol∙l^−1^ lower in our study compared to others in artistic gymnastics [9, 10, 25], even the highest reported values for FC in WAG of up to 8.5 mmol∙l^−1^ revealed submaximal BLC values [31]. Similarly, the BLC_peak_ values in MAG were well within the previously reported range of 6–7 mmol∙l^−1^ [7] and the lactic shares in WAG and MAG in our study were slightly higher than in 60 s simulated judo matches (9.9%) [27] and in figure skating (11.6%) [14]. However, the difficulty of elements in this study had to be slightly reduced because athletes wore the spirometry throughout the test. This is likely to influence absolute energetics and may also influence relative energy contribution. A higher difficulty may lead to higher jumps, more reactive forces and faster rotations which may affect total energy demand and/or anaerobic energy contribution.

All in all, the energy systems appear to be taxed submaximal in floor gymnastics. This seems reasonable in a sport where difficulty and execution of artistic elements are most decisive. The submaximal, non-decisive character is supported by no significant correlation between any metabolic parameter and FC score. This indicates that no energetic parameter directly influenced floor performance in this simulation study. However, anaerobic alactic share was correlated significantly with training experience and anaerobic lactic share with actual competition level. Finally, the FC score was correlated with the time competing at the respective performance category. This again supports the notion that training level plays an important role to execute artistic elements on a high level which is probably easier when the metabolic stress is at submaximal level for each energy system [13]. Moreover, aspects like technical execution may be closely related to postural control and muscle activation patterns which in turn may influence energy demand [32, 33]. However, from our perspective it seems hardly possible to assess these interrelations in an applied setting with the currently available methods. Therefore, we argue that floor gymnastics-specific metabolic share rather functions as an “admission ticket” to sports performance and should secure the execution of the routine by adequate energy supply. This may be of particular importance toward the end of the routine and regarding elements involving jumps or difficult combinations of single elements.

### Sex-Specific Differences of Energetics in Floor Gymnastics

Before analyzing and interpreting the differences in athletic performance and energetics between WAG and MAG it must be considered that sex may influence these aspects in two ways. Firstly, athletes compete in a category based on their biological sex. The two categories have different rules which do not only imply different competition durations but also different judgment rules [2, 3]. These in turn cause differences in the elements that are chosen, the way they are performed and combined. Explicitly, the current rules lead to a higher “fluency” in WAG. This in turn is likely to lead to different conditioning strategies. Secondly, sex may lead to differences in muscle metabolism and quantity [34]. Overall, athletic performance markers between female and male gymnasts were similar in most athletic tests when normalized to bodyweight [31]. However, the jumping tests of male athletes showed higher power values in CMJ and higher relative mean power in CJ30, while relative DJ power and relative peak power in CJ30 were not significantly different between male and female athletes. In this case, the effect sizes (see Table 1) indicate a trend toward higher relative values in male athletes. The reactive speed as measured by DJ ground contact time was equal in male and female athletes. While comparable ground contact times between sexes seem to be not uncommon in artistic gymnastics, the DJ ground contact time is on the upper threshold expected from female athletes for the Swiss national team [35]. DJ heights in this study were both on the lower end of the demands for Swiss female and male athletes, respectively [35]. Moreover, although FC time was significantly longer in WAG than in MAG metabolic energy was similar for WAG and MAG while metabolic power was significantly higher in MAG. In conclusion, this represents a higher relative power in male gymnasts which may be caused by a slightly higher relative muscle mass and a shorter competition duration leading to different conditioning patterns in WAG and MAG [31].

Relative energy contribution to FC was different between sexes in relative aerobic contribution, only. Meanwhile, no statistically significant differences were found for the relative anaerobic lactic and anaerobic alactic share, although the effect sizes may indicate trends toward lower anaerobic lactic and alactic shares in WAG. Since FC time was significantly different in WAG and MAG because of the official gymnastic rules [2, 3] the different relative aerobic energy contribution is not surprising. Although exercise intensity during FC seems to be submaximal to near-maximal the difference in aerobic contribution is in line with previous estimation models for continuous maximal exercise [11]. In addition to the different exercise time there is evidence that fiber type distribution may vary considerably between women and men with a higher proportion of type I fibers in women and a higher proportion of type IIA and IIX fibers in men [34]. A higher relative amount of type I fibers may also lead to a higher relative aerobic share. Due to methodological and ethical constraints, we were not able to assess fiber type distribution in our subjects which would have added evidence. However, not only FC time is different between WAG and MAG but also the creative or original movements, connections and transitions between acrobatic lines are different and likely more intense in WAG than MAG [2, 3]. This results in the need for women to perform in a more fluently manner and to combine the single artistic elements with more rhythmic elements compared to male athletes. Consequently, this may lead to smaller fluctuations in energy demand as there may be less parts in the routine where female athletes can “take a breath” and prepare for the next path, unlike this may be the case in MAG. The difference in competition demands may also play a role for a possible difference in anaerobic energy share in WAG and MAG. Relative anaerobic lactic energy contribution trended to be somewhat lower in WAG compared to MAG. This may be the result of a multifactorial difference caused by regulations and possible anthropometric differences between female and male gymnasts. Besides the shorter competition time in MAG there may also be more static elements inducing a higher energy demand in MAG compared to WAG. Moreover, a higher proportion of type IIA and type IIX in male than in female athletes [34] and a likely higher total muscle mass in male gymnasts may lead to a slightly higher anaerobic lactic energy contribution. The latter aspects may also influence the magnitude of the relative anaerobic alactic share. The anaerobic alactic system may mainly be taxed by jumps and reactive movements in both WAG and MAG. For both sexes the interindividual variations in this energy domain were relatively large (see Fig. 1), indicating a possible strong influence of individual physique and training status. Moreover, the trend toward a higher anaerobic alactic energy contribution in MAG may reflect the difference in FC time. As we only tested athletes of sub-elite level it is difficult to draw conclusions on how element difficulty and training level may affect absolute and/or relative energy contribution, although this has been shown for other sports [13, 36]. For floor routines we may assume that elements with higher difficulty could be more energy demanding because for example athletes need to jump higher and flip or rotate faster. On the other hand, energy share is influenced by biomechanical efficiency during stretch–shortening-cycle movements [19] and elite gymnasts likely possess better postural control during the same element [32, 33]. Therefore, these two mechanisms may in turn reduce energy demand and/or modify energy share for a certain element in athletes of higher execution level. Consequently, further research on the influence of competition level on energetics in gymnastics is currently warranted. All in all, the differences between WAG and MAG confirm the general notion of the national association that different conditioning strategies may be relevant for WAG and MAG [37, 38]. However, this should be further elucidated in future studies with higher sample size and different performance levels.

## Practical Applications

Based on the results of this study we recommend continuing the common sex-specific conditioning. In terms of specific “aerobic” training, routines consisting of basic elements may be implemented to be better prepared for competition duration. In addition, “mini routines” that are executed repeatedly with, e.g., 5–10 s breaks may improve the inter-effort recovery.

## Limitations

A major limitation of this study was that we could not assess possible influencing factors on relative energy share such as muscle fiber type distribution or total muscle mass. Also, we did not compare athletes of different competitive level. Despite the relatively small sample size our sub-elite athletes are likely to represent at least a certain spectrum of the above-named factors. We provide confidence intervals for all relative energy shares which provide an estimate for inter-individual differences within one energy share. Finally, the PCr-LA-O_2_ is an indirect method to assess energy contribution and uses several methodological assumptions, including the one that a high quality of fit is guaranteed for the EPOC kinetics. To achieve high methodological quality two experienced researchers screened the raw data and the goodness of fit (*r*^2^) for EPOC kinetics was kept above 0.95. Further information on the limitations of the PCr-LA-O_2_ can also be found in previous studies [39, 40]. These aspects need to be considered when the results of this study are interpreted or compared to other results.

## Conclusion

Although artistic gymnastics is generally considered to be an “anaerobic sport”, the relative anaerobic energy contribution is neither predominant in WAG nor MAG. However, anaerobic energy contribution plays a major role in both WAG and MAG, but both the anaerobic alactic and the anaerobic lactic system are taxed submaximal in WAG and MAG. In order to properly build up sports-specific endurance in gymnastics, training programs should strengthen sex-specific routines with a duration comparable to competition time as a form of sport-specific-endurance training.

## Data Availability

Data are available from the corresponding author on reasonable request.

## Abbreviations

ANOVA: Analysis of variance
BLC: Blood lactate concentration
CdP: Code de pointage
CI: Confidence interval
CJ30: 30S continuous jumping test (Bosco Test)
CMJ: Counter-movement jump
DJ: Drop jump
EPOC: Excess post-exercise oxygen consumption
FC: Floor competition
MAG: Men’s artistic gymnastics
MP: Mean power
NET-BLC / ∆BLC: Difference between peak and rest blood lactate concentration
PCr-LA-O_2_: Continuous version of the three-component-model for energy contribution modeling
PP: Peak power
*P*_tot_: Metabolic power
VO_2_: Oxygen uptake
VO_2EPOC_: Oxygen amount of the fast component of post-exercise oxygen consumption
VO_2PCr_: Amount of oxygen that is used for aerobic rephosphorylation
VO_2peak_: Peak oxygen uptake
WAG: Women’s artistic gymnastics
*W*_AER_: Aerobic energy
*W*_BLC_: Anaerobic lactic energy
*W*_PCr_: Anaerobic alactic energy
*W*_tot_: Metabolic energy
